# Updates on the Status of Vitamin D as a Risk Factor for Respiratory Distress Syndrome

**DOI:** 10.1155/2018/8494816

**Published:** 2018-09-30

**Authors:** Vesara Ardhe Gatera, Rizky Abdulah, Ida Musfiroh, Raden Tina Dewi Judistiani, Budi Setiabudiawan

**Affiliations:** ^1^Department of Pharmacology and Clinical Pharmacy, Faculty of Pharmacy, Universitas Padjadjaran, Bandung, Indonesia; ^2^Department of Pharmaceutical Analysis and Medicinal Chemistry, Faculty of Pharmacy, Universitas Padjadjaran, Bandung, Indonesia; ^3^Department of Public Health, Faculty of Medicine, Universitas Padjadjaran, Bandung, Indonesia; ^4^Department of Child Health, Faculty of Medicine, Universitas Padjadjaran, Bandung, Indonesia

## Abstract

To update the guidelines regarding vitamin D status in respiratory distress syndrome, we reviewed recent human and animal studies on the benefits of vitamin D in respiratory distress. We searched PubMed and ProQuest for studies on the use of vitamin D from 2009 to 2017. The common parameters in these studies included the use of lung tissue, phospholipids, blood, and plasma to assess the effects of vitamin D on respiratory syndrome. The metabolized form of vitamin D used in these studies was 1,25(OH)_2_D_3_ in animal studies and 25(OH)D in human studies. Vitamin D supplementation decreases the risk of respiratory distress syndrome, improves the quality of life, and is relatively effective and safe for preterm neonates as well as during lung maturation. However, although vitamin D supplementation may offer benefits for respiratory distress syndrome, the optimal dosing strategies for specific types of risk factors in the lungs must be clarified to confirm the therapeutic efficacy.

## 1. Introduction

Vitamin D has been reported to play roles in musculoskeletal function, regulation of hormone secretion, immune system function, and regulation of cell proliferation and differentiation [1]. In its classic function, small doses of vitamin D are necessary to regulate the calcium and phosphate balance, and it also plays an anti-inflammatory role. In addition, vitamin D receptor interacts with many chromosomes to effect different organs [2]. Vitamin D is naturally obtained from sunlight and synthesized by the body.

More recently, vitamin D has been shown to assist in lung maturation. The correlation between lung maturation and vitamin D is explained by the mechanism of phospholipid (surfactant) production and secretion on the surface of alveolar type II (ATII) cells [3]. The concentration of surfactant in ATII cells is associated with pregnancy gestation; therefore, the maturation of lung surfactant also progresses with pregnancy gestation.

Knowledge of the role of vitamin D has expanded over many years, and research has been conducted to determine the association between vitamin D status and health problems. American adult populations over the last 20 years have obtained only an estimated 40% of the necessary vitamin D levels. Vitamin D deficiency can increase the risk of many other diseases of the musculoskeletal, cardiovascular, and respiratory systems, as well as cancer [4–13]. However, more evidence is necessary to determine the association with these diseases, particularly for respiratory distress syndrome.

In 2015, Lykkedegn et al. reported evidence on the benefits of vitamin D for respiratory distress syndrome treatment and prevention. They showed substantial evidence of multiple physiological actions through which vitamin D stimulates maturation of the fetal lung, including ATII cell maturation and alveolarization. Their results supported the hypothesis that vitamin D deficiency or insufficiency is a frequent, modifiable risk factor for respiratory distress syndrome [3].

The recommended levels of vitamin D are divided into a few classifications as shown in Table 1. The recommendations for vitamin D levels require more evidence and research, particularly for the prevention or treatment of respiratory distress syndrome. The classifications from the Institute of Medicine [14], the Endocrine Society [15], and the Pediatric Endocrine Society [16] are not the final official recommendations. Therefore, more studies are necessary to demonstrate the correlation between vitamin D deficiency and respiratory distress syndrome.

Respiratory distress syndrome is known to be associated with the hyaline membrane. This disease can worsen and lead to atelectasis, hypoxia, hypercarbia, tachypnea, retraction, asthma, and pneumonia. For almost three decades, the primary treatment of this disease has been corticosteroids and exogenous surfactants, which come with many problems such as high cost and side effects, especially in children. Respiratory distress syndrome is also triggered by physical, chemical, and hormonal systems that influence the maturation and production of lungs and the secretion of other components such as phospholipids. Generally, respiratory distress syndrome occurs in premature babies during normal conditions or operation procedures [17].

Phospholipids can spread to other locations after entering the alveolar system, thus reducing the concentration of lung surfactant. The main cause of respiratory distress syndrome is the lack of surfactant in the lungs. This surfactant is primarily composed of phospholipids and is responsible for maintaining the surface tension of the lungs. Immaturity in the lung structure, coupled with low amounts of surfactant, is known to reduce the compliance and increase the propensity of other complications, such as atelectasis. Atelectasis induces slight ventilation of the lungs and prompts the mismatch of (V/Q), as well as alveolar hypoventilation such as hypercarbia and hypoxemia. Subsequent hypoxemia and hypoperfusion disrupt O_2_ circulation, leading to anaerobic metabolism and lactic acidosis. This condition impacts acidosis hypoxemia and increases pulmonary vasoconstriction. Other complications such as volutrauma, and high FIO_2_ may stimulate chemokines and cytokine inflammatory mechanisms, inducing injury to the epithelial cells. Epithelial cell injury reduces lung surfactant production and increases the permeability of the membrane, which can develop into complications such as respiratory distress syndrome [18].

## 2. Methodology

**Figure 1:**
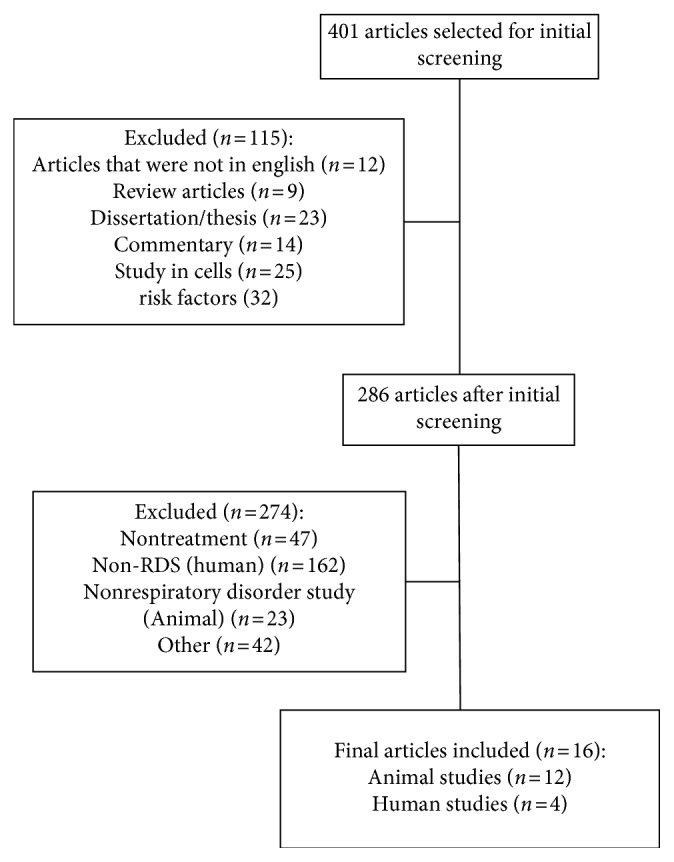
Flowchart of the literature search.

Our review is based on the literature obtained from the PubMed and ProQuest databases using the keywords “Vitamin D deficiency” and “Respiratory Distress Syndrome.” The specific keywords related to the pathophysiology of respiratory distress syndrome used were “Surfactant deficiency,” “atelectasis,” “cytokines,” and “pulmonary vasoconstriction.” The following exclusion criteria were applied: reviews, opinions or commentaries, non-English language, and unrelated topics such as the lack of vitamin D data, surfactant deficiency, and chronic respiratory syndrome disease. The flowchart of the literature search can be seen in Figure 1.

## 3. Animal Studies on the Roles of Vitamin D in Lung Development

Twelve animal studies on the roles of vitamin D in lung development were included in the current study (Table 2). Nguyen et al. performed a study on vitamin D and respiratory disease in 1987 using Wistar rats to determine the distribution of vitamin D in the last quarter of pregnancy. This study assessed the binding site of vitamin D. The results demonstrated that vitamin D not only functions in the skeleton but also in the lungs, particularly the metabolite of vitamin D known as 1,25(OH)_2_D_3_. Then they further investigated the potential role of vitamin D in respiratory distress syndrome in 1990 by using Sprague-Dawley rats to identify the location of the vitamin D receptor. The results showed that the receptor was not located in ATII cells during the postnatal period. The mechanism by which vitamin D in its metabolite form (1,25(OH)_2_D_3_) appears in the receptor, however, remains unclear [31, 32].

In the same year, research from Marin et al. confirmed that the vitamin D metabolite (1,25(OH)_2_D_3_) increased the presence of phospholipids, which are a primary component of surfactants. They found that dexamethasone could also increase the presence of phospholipids, but the mechanism was unclear. Conversely, the secosteroid vitamin D has been shown to have the same mechanism as dexamethasone when administered for respiratory distress syndrome treatment [33].

As the mechanism of vitamin D in ATII cells remains unclear, in 1993, Edelson et al. attempted to answer this question by using an animal model to determine the role of 1,25(OH)_2_D_3_ in ATII cells. The results showed that 1,25(OH)_2_D_3_ had the potential to bind in ATII cells, but only in neonatal and adult epithelial cells [34]. Similarly, in 2009, Sakurai et al. explored this question through a biomolecular approach. They evaluated cell proliferation, fibroblast apoptosis, alveolar epithelial-mesenchymal interactions, and morphology with Sprague-Dawley rat models using various doses (1,25D (10 ng/kg body weight) or 3-epi-1,25D (50 ng/kg body weight) administered once daily intraperitoneally, starting from the day of delivery (day 0). The data showed that supplementation of vitamin D in its active metabolite form could increase the synthesis of surfactants during the maturation of the lungs [19].

In 2011, Zosky et al. employed another approach to investigate the mechanism of vitamin D with various intervention doses in newborn BALB/c mice with the aim to determine the development of lung volume (TGV), lung mechanics, and lung structure. In this study, they found a correlation between vitamin D deficiency and lung maturation, supporting the association of vitamin D status with lung disease [20]. In 2014, Yurt et al. furthered Zosky's work on lung morphology, phospholipid synthesis, alveolar epithelial-mesenchymal interactions, and lung function. They used Sprague-Dawley rats to determine the optimal time for vitamin D supplementation during pregnancy. The results showed that vitamin D deficiency influences alveolar cells and airway contractility. Vitamin D supplementation improved lung function at a dose of 500 IU/kg in a rat model [21].

Vitamin D deficiency in lung injury was also reported to exaggerated alveolar inflammation, epithelial damage, and hypoxia of lipopolysaccharide- (LPS-) induced acute lung injury [22]. Furthermore, it was also found that the lack of vitamin D receptor in the pulmonary epithelial barrier will lead to a more severe LPS-induced lung injury [23]. The protective role of vitamin D in LPS-induced lung injury was then explained by report of Xu et al. to be through regulation of the balance between the expression of members of the renin-angiotensin system [24]. Conflicting result, however, also found by Klaff et al. that reported LPS-induced lung injury was independent of serum vitamin D concentration [25].

To confirm the influence of modified doses of vitamin D, Mandell et al. conducted another study in 2014 using Sprague-Dawley rats fed a diet including vitamin D supplements. The study evaluated the optimal time and showed that early treatment was better because it could improve alveolar maturation by 14% in 14 days after birth [26]. Similar to the studies on vitamin D deficiency, in 2016, Lykkedegn et al. evaluated vitamin D depletion and the development of lung surfactant in female Sprague-Dawley rats. The animal models were used to compare two groups with different doses: a diet group (*n*=18) (<5 IU/kg cholecalciferol) and a control diet group (*n*=16) (1500 IU/kg cholecalciferol). This study confirmed the association between vitamin D depletion and respiratory insufficiency in preterm neonates [27].

In 2015, Chen et al. confirmed the association between high vitamin D levels in the form of 1,25(OH)2D3 on interleukin (IL)-4, IL-12, IL-13, interferon-γ, and ovalbumin-JAK1/p-STAT6/SOCS5. The results showed that the level of vitamin D was related to many pathological aspects of respiratory distress at the molecular level because it influenced the amount of specific proteins in ATII cells [28]. In another study on the molecular level, particularly regarding protein expression and vitamin D deficiency, Chen et al. identified the lung development pathways that are sensitive to vitamin D deficiency using female BALB/c mouse models in 2016. The study involved control mice that were vitamin D-deficient (no vitamin D3 intake). The treatment mice were fed replete (2195 IU/kg vitamin D3) diets (Specialty Feeds, Glen Forrest, Western Australia) for at least 5 weeks before mating with the replete male mice. The results showed that there was no difference in protein expression in the offspring [29]. To investigate the optimal dosing method, Taylor et al. in 2016 provided vitamin D treatment through inhalation with the goal of improving safety, especially during lung maturation for the prevention of respiratory distress syndrome. Metabolites of vitamin D 1,25(OH)_2_D_3_ and 25(OH)_2_D_3_ were fed to rats through nebulization daily for 14 days. The extent of lung maturation and the serum vitamin D levels were determined by analysis of the pups, lungs, kidneys, and blood. Nebulized 1,25(OH)_2_D_3_ and 25(OH)_2_D_3_ increased lung maturation by improving the protein marker expression of lung epithelial cells (SP-B, leptin protein), as well as mesenchymal (PPARγ, C/EBP*α*) and endothelial (VEGF, FLK-1) cell differentiation, surfactant phospholipid synthesis, and lung morphology. However, the data indicated insignificant increases in 25(OH)_2_D_3_ [30].

## 4. Clinical Studies on Vitamin D as a Risk Factor for Respiratory Distress Syndrome

Four studies on the roles of vitamin D as a risk factor for respiratory distress syndrome are included in the current review (Table 3) [22,35–37]. Glasgow et al. in 1977 analyzed four subjects who received vitamin D intervention with different clinical outcomes and suggested that vitamin D contributed to the prevention of complications in preterm babies with respiratory distress [38].

The level of vitamin D in preterm babies with respiratory distress was evaluated by Ataseven et al. in 2013. Peripheral blood samples were obtained from 150 preterm newborns, born after 29–35 weeks of gestation, to investigate whether 25(OH)D deficiency is a risk factor for respiratory distress syndrome. In this study, no correlation between gestational age and vitamin D levels was found. However, the incidence of respiratory distress syndrome was significantly higher in preterm babies in severe condition (28%). High levels of vitamin D reduced the risk of respiratory distress syndrome by 3.34 times [35].

Another study on vitamin D levels was conducted by Dancer et al. in 2015. They showed that vitamin D deficiency is a common problem, particularly in patients with respiratory distress syndrome. In vitro studies have shown that vitamin D deficiency leads to inflammation in epithelial cells and affects more than 600 genes. These in vivo and in vitro studies demonstrate that vitamin D levels influence the risk of acute respiratory distress syndrome. However, they suggest that other risk factors related to this situation should be examined [22].

The association between vitamin D levels and respiratory distress syndrome was also assessed by Barnett et al. in 2013. The authors showed that the level of 25-OHD measured early after admission to intensive care was not associated with the development of acute lung injury and hospital or one-year mortality in critically ill patients with sepsis. However, lower 25-OHD levels were associated with higher one-year mortality in patients with severe trauma [36].

## 5. Conclusions and Future Research

This review paper summarizes 11 studies on vitamin D and risk factors conducted between 2009 and 2016. The studies included animal and human patients and assessed vitamin D status and its many associations with ATII cells. This review highlights the sparse data on many factors, particularly doses. The tables presented above show research in animals with many different contexts, but further research is necessary both in humans and in animals. Vitamin D deficiency affects lung structure and function by lowering oxygenation and reducing the survival time in preterm neonates. In addition, vitamin D can effectively improve lung maturation, lung volume, and ATII cells; it can also induce respiratory distress syndrome in preterm neonates. In future studies, the optimal time periods for vitamin D supplementation should be further explored to determine the relationship with prophylaxis or treatment.

The results of our summary suggest the benefits of vitamin D supplementation at doses of 250–1000 IU/kg in animals. More evidence-based information and additional studies are necessary to confirm the therapeutic benefits of vitamin D supplementation in humans and animals and to describe the molecular pathways of these mechanisms. Furthermore, more evidence is necessary to determine the best therapeutic use of vitamin D, particularly regarding doses, optimal time, protein expression, and toxicity in humans and animals. Additionally, the optimal dosing strategies for specific types of risk factors in the lungs must be clarified to confirm the therapeutic efficacy before it can be recommended for broad clinical use.

## Figures and Tables

**Table 1:** Recommendations for vitamin D level in human.

No	Vitamin D level	Institute of medicine classification [14]	Endocrine society classification [15]	Pediatric endocrine society classification [16]
1	Toxicity (ng/mL)	—	>150	>150
2	Risk of toxicity (ng/mL)	>50	—	>100
3	Insufficiency (ng/mL)	—	21–29	15–20
4	Sufficiency (ng/mL)	<20	>30	20–100
5	Deficiency (ng/mL)	<15	<20	<15
6	Severe deficiency (ng/mL)	<5	—	<5

**Table 2:** Reports of the correlation between vitamin D deficiency and respiratory distress syndrome in animal studies.

No	References	Animals	Doses	Main findings
1	Sakurai et al. 2009 [19]	Sprague-Dawley rats	1,25D (10 ng/kg body wt) or 3-epi-1,25D (50 ng/kg body wt) once daily	Vitamin D works in the lung maturation of perinatal rats
2	Zosky et al. 2011 [20]	Newborn BALB/c mice	(2,195 IU/kg) diets (Specialty Feeds, Glen Forrest, Western Australia), (500 ng/g)	Vitamin D deficiency decreased the lung volume
3	Yurt et al. 2014 [21]	Sprague-Dawley rats	250 IU/kg cholecalciferol (no. 1814547), 500 IU/kg cholecalciferol (no. 1814548), 1,000 IU/kg cholecalciferol (no. 1814549)	Vitamin D at 500 IU/kg effectively blocks the deficiency of VD
4	Dancer et al. 2015 [22]	Wild-type (WT) C57Bl/6	—	Vitamin D deficiency contributes directly to the acute respiratory distress syndrome (ARDS)
5	Shi et al. 2016 [23]	Mice with a C57BL/6J	—	Vitamin D/VDR signaling attenuates lipopolysaccharide-induced acute lung injury by maintaining the integrity of the pulmonary epithelial barrier
6	Xu et al. 2017 [24]	Wistar rats	1, 5 or 25 mg/kg calcitriol	Vitamin D alleviates lipopolysaccharide-induced acute lung injury via regulation of the renin-angiotensin system
7	Klaff et al. 2012 [25]	C57BL/6 mice	1000 IU/kg of cholecalciferol	Found no difference in the degree of lung injury
8	Mandell et al. 2014 [26]	Sprague-Dawley rats	(500 ng/g)	Vitamin D increased alveolar type II cells (ATII) in fetal rats
9	Lykkedegn et al. 2016 [27]	Female Sprague-Dawley rats	<5 IU/kg of cholecalciferol, 1500 IU/kg of cholecalciferol	Vitamin D deficiency induces lower oxygenation and reduces the survival time in preterm rats
10	Chen et al. 2015 [28]	Male Wistar rats	Vitamin D at 4, 1, 4, and 10 μg/kg	Vitamin D works to block airway inflammation
11	Chen L et al. 2016 [29]	Female BALB/c mice	(0 vitamin D3). (2195 IU/kg vitamin D3) diets (Specialty Feeds, Glen Forrest, Western Australia)	Vitamin D deficiency may influence lung structure and function in early postnatal mice
12	Taylor et al. 2016 [30]	Sprague-Dawley rats	25(OH) D subdivided into 100, 500, or 1000 ng/kg. 1,25D, subdivided into 1, 10, or 50 ng/kg	Vitamin D is relatively safe and effective in the lung maturation of neonatal rats

**Table 3:** Reports of the correlation between vitamin D deficiency and respiratory distress syndrome in human studies.

No	Source	Population	Results
1	Ataseven et al. 2013 [35]	Preterm newborns, born at 29–35 weeks gestational age	Respiratory distress syndrome was more common in preterm babies with 25(OH)D deficiency
2	Barnett et al. 2013 [36]	Patients with sepsis and trauma with or without ALI/ARDS	25-OHD levels did not differ between cases with ALI/ARDS
3	Dancer et al. 2015 [22]	Human (primary alveolar epithelial cells)	Vitamin D has effects on primary human alveolar epithelial cells affecting >600 genes
4	Fettah et al. 2015 [37]	81 preterm infants	Lower cord blood 25(OH)D levels might be associated with increased risk of RDS in preterm infants with very low birth weight

## References

[1] Bikle D. (2009). Nonclassic actions of vitamin D. *Journal of Clinical Endocrinology and Metabolism*.

[2] Pludowski P., Holick M. F., Pilz S. (2013). Vitamin D effects on musculoskeletal health, immunity, autoimmunity, cardiovascular disease, cancer, fertility, pregnancy, dementia and mortality-a review of recent evidence. *Autoimmunity Reviews*.

[3] Lykkedegn S., Sorensen G. L., Beck-Nielsen S. S., Christesen H. T. (2015). The impact of vitamin D on fetal and neonatal lung maturation. A systematic review. *American Journal of Physiology-Lung Cellular and Molecular Physiology*.

[4] Forrest K. Y., Stuhldreher W. L. (2011). Prevalence and correlates of vitamin D deficiency in US adults. *Nutrition Research*.

[5] Ginde A. A., Liu M. C., Camargo C. A. (2009). Demographic differences and trends of vitamin D insufficiency in the US population, 1988-2004. *Archives of Internal Medicine*.

[6] Holick M. F. (2006). Resurrection of vitamin D deficiency and rickets. *Journal of Clinical Investigation*.

[7] Visser M., Deeg D. J., Lips P. (2003). Low vitamin D and high parathyroid hormone levels as determinants of loss of muscle strength and muscle mass (sarcopenia): the longitudinal aging study Amsterdam. *Journal of Clinical Endocrinology and Metabolism*.

[8] Bischoff H. A., Stähelin H. B., Dick W. (2003). Effects of vitamin D and calcium supplementation on falls: a randomized controlled trial. *Journal of Bone and Mineral Research*.

[9] Wang T. J., Pencina M. J., Booth S. L. (2008). Vitamin D deficiency and risk of cardiovascular disease. *Circulation*.

[10] Lappe J. M., Travers-Gustafson D., Michael Davies K., Recker R. R., Heaney R. P. (2007). Vitamin D and calcium supplementation reduces cancer risk: results of a randomized trial. *American Journal of Clinical Nutrition*.

[11] Laaksi I., Ruohola J.-P., Tuohimaa P. (2007). An association of serum vitamin D concentrations < 40 nmol/L with acute respiratory tract infection in young Finnish men. *American Journal of Clinical Nutrition*.

[12] Gibney K. B., MacGregor L., Leder K. (2008). Vitamin D deficiency is associated with tuberculosis and latent tuberculosis infection in immigrants from sub-Saharan Africa. *Clinical Infectious Diseases*.

[13] Ginde A. A., Mansbach J. M., Camargo C. A. (2009). Association between serum 25-hydroxyvitamin D level and upper respiratory tract infection in the third national health and nutrition examination survey. *Archives of Internal Medicine*.

[14] Del Valle H. B., Yaktine A. L., Taylor C. L., Ross A. C., Institute of Medicine (2011). *Dietary Reference Intakes for Calcium and Vitamin D*.

[15] Holick M. F., Binkley N. C., Bischoff-Ferrari H. A. (2011). Evaluation, treatment, and prevention of vitamin D deficiency: an endocrine society clinical practice guideline. *Journal of Clinical Endocrinology and Metabolism*.

[16] Misra M., Petryk A., Collett-Solberg P. F. (2008). Vitamin D deficiency in children and its management: review of current knowledge and recommendations. *Pediatrics*.

[17] Mercer B. M., Creasy R. K., Resnik R., Lockwood C. J., Greene M. F., Moore T. (2009). Assesement and induction of fetal pulmonary maturity. *Creasy & Resnik’s Maternal-Fetal Medicine*.

[18] Locci G., Fanos V., Gerosa C., Faa G. (2014). Hyaline membrane disease (HMD): the role of the perinatal pathologist. *Journal of Pediatric and Neonatal Individualized Medicine*.

[19] Sakurai R., Shin E., Fonseca S. (2009). 1alpha,25(OH)2D3 and its 3-epimer promote rat lung alveolar epithelial-mesenchymal interactions and inhibit lipofibroblast apoptosis. *American Journal of Physiology-Lung Cellular and Molecular Physiology*.

[20] Zosky G. R., Berry L. J., Elliot J. G., James A. L., Gorman S., Hart P. H. (2011). Vitamin D deficiency causes deficits in lung function and alters lung structure. *American Journal of Respiratory and Critical Care Medicine*.

[21] Yurt M., Liu J., Sakurai R. (2014). Vitamin D supplementation blocks pulmonary structural and functional changes in a rat model of perinatal vitamin D deficiency. *American Journal of Physiology-Lung Cellular and Molecular Physiology*.

[22] Dancer R. C., Parekh D., Lax S. (2015). Vitamin D deficiency contributes directly to the acute respiratory distress syndrome (ARDS). *Thorax*.

[23] Shi Y. Y., Liu T., FU J. (2016). Vitamin D/VDR signaling attenuates lipopolysaccharideinduced acute lung injury by maintaining the integrity of the pulmonary epithelial barrier. *Molecular Medicine Reports*.

[24] Xu J., Yang J., Chen J., Luo Q., Zhang Q., Zhang H. (2017). Vitamin D alleviates lipopolysaccharideinduced acute lung injury via regulation of the reninangiotensin system. *Molecular Medicine Reports*.

[25] Klaff L. S., Gill S. E., Wisse B. E. (2012). Lipopolysaccharide-induced lung injury is independent of serum vitamin D concentration. *PLoS One*.

[26] Mandell E., Seedorf G., Gien J., Abman S. H. (2014). Vitamin D treatment improves survival and infant lung structure after intra-amniotic endotoxin exposure in rats: potential role for the prevention of bronchopulmonary dysplasia. *American Journal of Physiology-Lung Cellular and Molecular Physiology*.

[27] Lykkedegn S., Sorensen G. L., Beck-Nielsen S. S. (2016). Vitamin D depletion in pregnancy decreases survival time, oxygen saturation, lung weight and body weight in preterm rat offspring. *PLoS One*.

[28] Chen X., Rao S. Q., Gao B. H., Jiang Z. Q. (2015). Effect of early vitamin D supplementation on asthma and the possible mechanisms. *Genetics and Molecular Research*.

[29] Chen L., Wilson R., Bennett E., Zosky G. R. (2016). Identification of vitamin D sensitive pathways during lung development. *Respiratory Research*.

[30] Taylor S. K., Sakurai R., Sakurai T., Rehan V. K. (2016). Inhaled vitamin D: a novel strategy to enhance neonatal lung maturation. *Lung*.

[31] Nguyen M., Guillozo H., Garabédian M., Balsan S. (1987). Lung as a possible additional target organ for vitamin D during fetal life in the rat. *Neonatology*.

[32] Nguyen T. M., Guillozo H., Marin L. (1990). 1,25-dihydroxyvitamin D3 receptors in rat lung during the perinatal period: regulation and immunohistochemical localization. *Endocrinology*.

[33] Marin L., Dufour M. E., Tordet C., Nguyen M. (1990). 1,25(OH)2D3 stimulates phospholipid biosynthesis and surfactant release in fetal rat lung explants. *Neonatology*.

[34] Edelson J. D., Chan S., Jassal D., Post M., Tanswell A. K. (1994). Vitamin D stimulates DNA synthesis in alveolar type-II cells. *Biochim Biophys Acta*.

[35] Ataseven F., Aygün C., Okuyucu A., Bedir A., Kücük Y., Kücüködük Ş. (2013). Is vitamin d deficiency a risk factor for respiratory distress syndrome?. *International Journal for Vitamin and Nutrition Research*.

[36] Barnett N., Zhao Z., Koyama T. (2014). Vitamin D deficiency and risk of acute lung injury in severe sepsis and severe trauma: a case-control study. *Annals of Intensive Care*.

[37] Fettah N. D., Dilli D., Beken S., Okumuş N., Fettah N. (2015). Is higher 25-hydroxyvitamin D level preventive for respiratory distress syndrome in preterm infants?. *American Journal of Perinatology*.

[38] Glasgow J. F., Thomas P. S. (1977). Rachitic respiratory distress in small preterm infants. *Archives of Disease in Childhood*.

